# On the Lack of Consensus over the Meaning of Openness: An Empirical Study

**DOI:** 10.1371/journal.pone.0023420

**Published:** 2011-08-17

**Authors:** Alicia M. Grubb, Steve M. Easterbrook

**Affiliations:** Department of Computer Science, University of Toronto, Toronto, Canada; University of Modena and Reggio Emilia, Italy

## Abstract

This study set out to explore the views and motivations of those involved in a number of recent and current advocacy efforts (such as open science, computational provenance, and reproducible research) aimed at making science and scientific artifacts accessible to a wider audience. Using a exploratory approach, the study tested whether a consensus exists among advocates of these initiatives about the key concepts, exploring the meanings that scientists attach to the various mechanisms for sharing their work, and the social context in which this takes place. The study used a purposive sampling strategy to target scientists who have been active participants in these advocacy efforts, and an open-ended questionnaire to collect detailed opinions on the topics of reproducibility, credibility, scooping, data sharing, results sharing, and the effectiveness of the peer review process. We found evidence of a lack of agreement on the meaning of key terminology, and a lack of consensus on some of the broader goals of these advocacy efforts. These results can be explained through a closer examination of the divergent goals and approaches adopted by different advocacy efforts. We suggest that the scientific community could benefit from a broader discussion of what it means to make scientific research more accessible and how this might best be achieved.

## Introduction

Over the past quarter century, the amount of information available to society has grown dramatically. People now expect instant access to a variety of information about products, people, politics, and financial markets. However, the details of scientific research are a notable exception, as the majority of scientific artifacts are not freely available to the general public. Most scientific publications are available only through subscriptions, and even then it is rare that the supporting evidence (e.g., raw data, statistical analysis, algorithms, and results) is available at all. Scientific results therefore filter through to the public via mainstream media, opening them up to distortion. The lack of access to scientific artifacts is sometimes interpreted as an indication that scientists have something to hide, thus decreasing their credibility.

Publishing in a respectable peer-reviewed journal has been the standard approach for scientists to communicate new results for over a century [1]. For academic researchers, such publications are essential for promotion, and to secure grants to enable future research. However, the increasing cost of access to scholarly journals has provoked talk of a crisis in scientific publishing (e.g. see [2] for a detailed analysis). Many commentators look to electronic publishing to reduce costs [3], and open access journals to remove the barriers to access [4].

In parallel with these trends in electronic publishing, some scientists have begun to call for greater reproducibility (e.g. see [5]), greater sharing of the intermediate stages of research (e.g. see the open science initiative [6]), and better capture of data provenance (e.g. see the provenance challenge [7]). However, there is as yet no consolidated effort among the advocates for these various initiatives.

All of these initiatives share a concern for more openness and better accountability of scientific artifacts. However, they also tend to assume that there is a broad consensus across the scientific community that their goals are the right ones, and that the main issues are to do with how we achieve them. To test whether such a consensus exists, this study set out to identify and categorize the views of scientists on the topics of reproducibility, credibility, scooping, data sharing, results sharing, and the effectiveness of the peer review process. We focussed primarily on the views of scientists who are advocates for these initiatives.

The study collected qualitative data through a free-form questionnaire consisting of 20 questions. The questionnaire was made available at two workshops devoted to discussion of issues around the interactions between science and the general public, and was also available online, in the summer of 2009, and advertised to these communities. The sampling approach was therefore deliberately purposive–we particularly targeted the data collection at scientists who are likely to identify with these initiatives in open science (see Methods).

We received nineteen responses to the questionnaire. The detailed qualitative responses provided insights into the respondents' reasoning processes as well as the terminology they used (see Methods). We used a number of different techniques to analyze this data. Some questions lent towards an iterative qualitative analysis based on grounded theory [8], in which the responses were used to derive representative terms that were grouped and further analyzed. Other questions enabled clustering of responses on numerical scales. The individual analysis process for each question is discussed in the Results section along side the findings. The limitations of this methodology are discussed later in this paper.

Our results can be categorized into four main themes: data and results sharing, peer review, the role of experiments and replications in science, and public perception of science. The study revealed a remarkable lack of consensus on many of these issues, and even a lack of shared terminology.

## Methods

To investigate how scientists think about issues of openness, we adopted a constructivist approach. Our aim is to explore the meanings that scientists attach to the various mechanisms for sharing their work, and the social context in which this takes place. As there are no existing theoretical accounts of the key phenomena involved in open science, our approach is explorative. It would be premature, for example, to seek to generalize scientists' opinions through quantifying responses to a large-scale survey instrument, as it is not clear what categories might be used for such a quantification, nor even whether a shared set of categories exists across the community. Hence, for this study, we focus on the collection of a rich set of qualitative data from a smaller number of participants, to explore their perspectives in detail.

We designed a set of 18 open-ended questions, along with 2 initial questions asking the scientists to identify their field of research, and ending with a further 7 questions seeking basic demographic information from participants (see Appendix S1). The open-ended questions span six topics: public perceptions of science, replications, the peer review process, results sharing, data sharing, and scooping. Care was taken to seek the respondents' own definitions of key terms, and to solicit examples for clarification where possible.

Subjects were recruited using a mix of random and purposive sampling. Our overall target population is *experimental scientists*, which we interpret to mean any scientist who performs experimental investigations of testable hypotheses, in which conditions are set up to isolate the variables of interest and test their effect on certain measurable outcomes. The research fields this covers are quite diverse, so we generalized the wording of the questionnaire as much as possible to suit any kind of experimental scientist. Our primary concern was to recruit scientists who have been active as advocates for each of three existing initiatives that seek to challenge conventional approaches to science and science communication, namely *computational provenance*, *reproducible research*, and *open science*. We discuss each of these initiatives in more detail in the Discussion section. We also sought scientists from outside these three initiatives, for comparison. We made no attempt to sample across *all* relevant fields of experimental science; rather our aim was to sample from communities associated with particular positions on openness in scientific research. We presume that opinions vary more widely in the general science community than in the openness communities. We do *not* attempt to identify how our findings might generalize to the greater science community.

Study participants were recruited at two workshops, SciBarCamp and Science 2.0. SciBarCamp, held in Palo Alto, California, was a free two-day conference focused around science and humanity. Approximately half of the attendees were from the San Francisco Bay area with the rest from other parts of the United States, Canada, the United Kingdom, and New Zealand. Science 2.0, held in Toronto, Ontario, was a free one day conference co-scheduled with a course in software design for computational scientists. The participants were mainly from Ontario with a few speakers from the United States and the United Kingdom. As the survey was conducted online, there may have been participants who did not attend either event, but were alerted to the questionnaire by one of the attendees. We also gave attendees at these two events the option to complete the questionnaire on paper; two participants chose this option with the rest completing the questionnaire online. We re-keyed the paper-based responses using the same format as the electronic responses.

In total, 30 responses were collected online and in-person. Of these responses, eleven were removed from the dataset prior to analysis, because the respondents did not answer any of the open questions. There are at least three possible explanations for this: (1) responses created by computer programs searching the internet; (2) respondents wanting to see the entire questionnaire before deciding to complete it; (3) respondents who abandoned the survey, perhaps realizing they did not meet the selection criteria. Three of the nineteen remaining online responses completed less than twenty percent of the questionnaire. These responses remained part of the data set but were only analyzed in the part where the questions were completed. The respondents consisted mostly of biologists (7), life scientists (4), chemists (3), and physicists (2). Table 1 give a complete list of respondents' representative field. The most represented sub-field was molecular biology with three respondents.

**Table 1:** Respondents by Research Field.

Research Field	Count
Applied Science	0
Astronomy	0
Biology	7
Chemistry	3
Earth Science	0
Environmental Science	0
Life Science	4
Medicine	1
Physics	2
Psychology	1
Other	1

The complete list of respondents' representative research field.

This study was reviewed and received ethics clearance through the Ethics Review Office at the University of Toronto. As in the spirit of this work, we would prefer to make both our results and analysis open and available. However, as a result of the strict rules for human subject ethics clearance, we cannot release our data or analysis. Furthermore, as detailed in our ethics clearance and subject consent letter we must ensure that:

- all respondent participation was voluntary;
- all information provided by respondents be kept confidential;
- all personal identifiers were stripped from the results before storage;
- individual responses could not be identified from the summarized data published;
- no data was released outside the approved research team.

## Results

The following sections describe the key findings of our questionnaire for each cluster of questions.

### Public Perception and Understanding of Science

Our study shows that scientists have conflicting views of how the general public perceives their work, although all respondents tend to believe the public does not have good knowledge of their work. Two questions directly tackled public perception: Q-3 (“How do you think the general public (non-scientists) views your particular field's research efforts?”) and Q-4 (“How do you think the general public (non-scientists) views the efforts of the scientific community as a whole?”). Q-3 examines the respondent's specific research field, whereas Q-4 examines the field of science in general.

We used iterative, open coding [8] to identify themes in the participants' responses to these two questions, with a set of representative terms emerging from the analysis. For example, “unaware” was selected as a representative term for the response below:

“Generally are not aware that basic research is going on in this area.”

The open coding generated fifteen representative terms for the responses to Q-3: apprehension, blind believers, boring, fear, generally positive, ignorance, irrelevant, ivory tower, misunderstanding, mysterious and complicated, obscure, positive, unaware, vaguely useful, and worthwhile.

**Figure 1:**
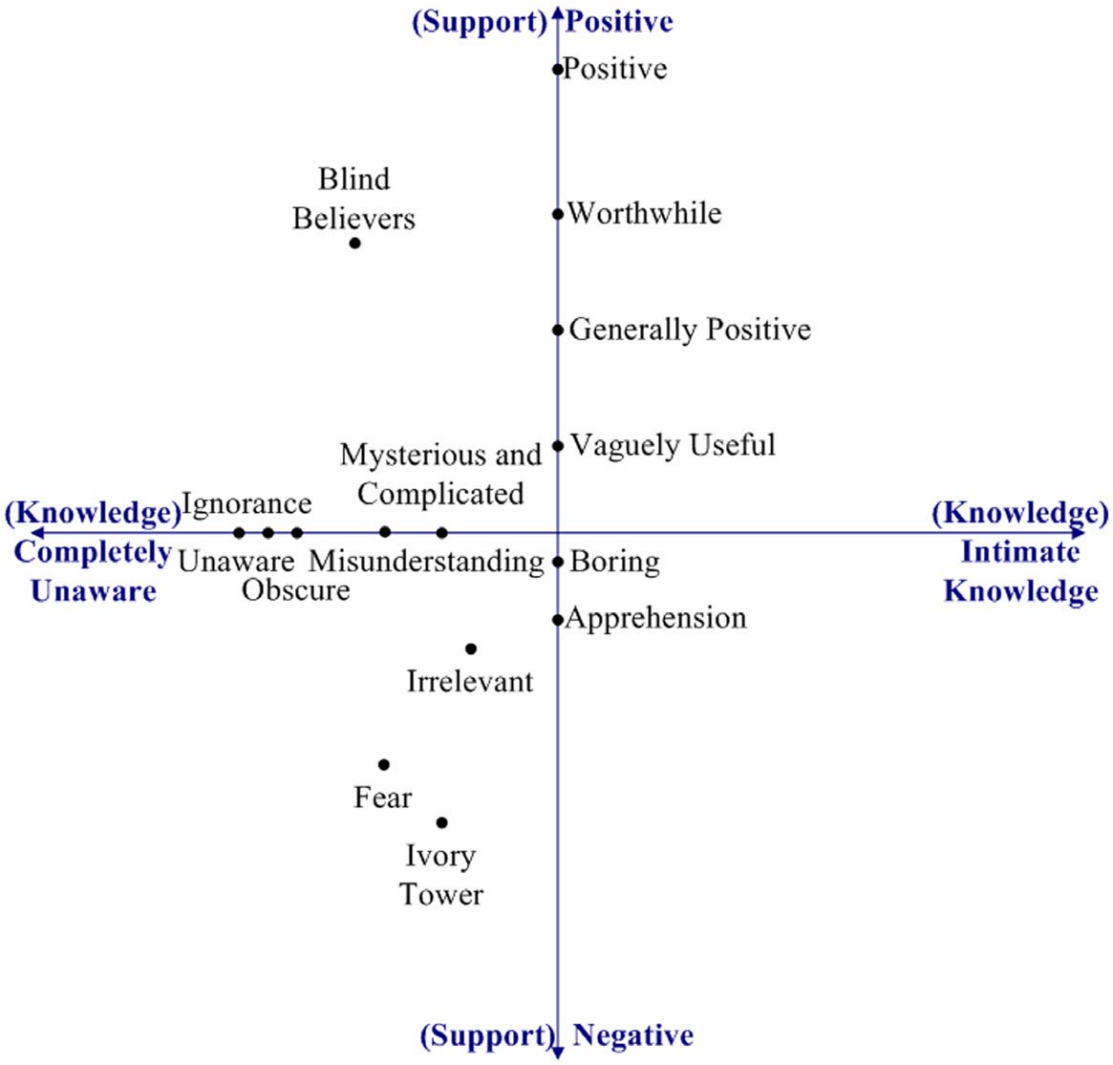
Plot for Q-3. Major emergent axes (*knowledge* and *support*) from Q-3 “How do you think the general public (non-scientists) views your particular field's research efforts?”.

We then applied axial coding [8] to identify conceptual axes to identify relationships between these terms. Two major axes emerged from this analysis: *knowledge* and *support* (see Figure 1). We developed the following interpretation of these two axes:

#### Support

Is a scale assessing level of approval and encouragement. We chose the midpoint to represent a neutral stance, or *indifference*.

#### Knowledge

Is a scale ranking the level of information and understanding of that information a person has. We chose the midpoint to represent a basic understanding.

We placed the representative terms on these axes, using our judgment of the original responses in the questionnaire. Since some terms capture ideas from both axes, we present the result on a two dimensional plot. The exact position of each term is a subjective judgment, but the plot is sufficient to identify some general patterns and clusters.

For the support axis, the responses spread across the full scale, indicating a diverse set of opinions from the respondents about how much the public supports their individual fields. However, for the knowledge axis, all responses were negative or neutral. This is interesting because it indicates that none of the respondents believe that people outside their field have a good knowledge of their work.

The participants' responses to Q-4 were much more diverse than for Q-3. Open coding of Q-4 responses generated thirty representative terms. When we clustered these, we ended up with four axes: engagement, knowledge, subject variability, and support. For knowledge and support we used the same axes as Q-3. The engagement axis is similar to the support axis, with impartiality as a midpoint. For subject variability, we clustered the terms describing subjects that were associated with positive support and ones that were associated with negative support. The four groups with their representative terms (in no specific order) are shown in Table 2.

**Table 2:** Q-4 Axes.

**engagement**	
positive:	awe, useful
negative:	caution, uninformed passion, unaware, boring, abstract, social misfits
**knowledge**	
positive:	
negative:	unaware, uninformed, can't understand details
**support**	
positive:	positive, useful, and important
negative:	bad, dangerous, suspicious motives, mistrust
**subject variability**	
positive:	medicine, cure, space, human impact
negative:	boring research, fruit flies

Major emergent axes (*knowledge*, *support*, *subject variability* and *engagement*) from Q-4 “How do you think the general public (non-scientists) views the efforts of the scientific community as a whole?”.

Breaking down the groups in the positive/negative sides, Table 2 reveals conflicting views among the respondents, with the only obvious area of agreement being a perception of the public as not knowledgeable (a similar response to Q-3). They perceived that there was little positive engagement, with some exceptions, while the support axis appears to be well balanced with both positive and negative viewpoints. The subject variability explains some of the diversity in responses about public engagement and support - the perception is that public opinion varies depending on the field of science under discussion. Fields of science that people found personally salient were discussed in more detail, and several of the respondents talked about current discussion of these fields in the media. Examples include:

“[W]ho cares about whether jellyfish change the mixing of the ocean when they swim around? Solve problems that matter to humanity.”

“[F]ields that cannot mention words like “cancer” or “HIV in infants” … are probably viewed with much less approval[.]”

Three responses mentioned Sarah Palin and “fruit fly research” as variations in subject matter, referring to Palin's comments mocking the funding of research on insects during the 2008 presidential election [9].

Overall, there is evidence of consensus among the respondents that the public lacks a good understanding of scientific research, both of science in general and of their own fields in particular. There is also some consensus that public support and engagement varies depending on the field, although we don't have sufficient data to determine whether respondents would agree on which fields these would be.

### Experiments, Publications, and Replications

#### Experiments

Q-5 asked respondents to explain what constitutes an experiment in their field. Respondents all used similar language when defining the term, but differed greatly in both in how they phrased their definition, and the scope of what counts a single experiment. Some definitions were more abstract such as:

“[a]n experiment is a planned series of measurements showing the effect of some treatment on an organism, by comparison to appropriate controls.”

Some were very specific to the subject domain such as:

“an experiment consists [of][,] for example[,] setting up a chemical reaction: context, thinking, literature research, preparing the glassware, ordering the chemicals, calculations, carrying out the whatever (cold/hot/inert etc), monitoring, working-up (preparation for that), isolation (e.g. chromatography with all the related things), drying or whatever is suitable, analysis experiments (e.g. NMR, optical rotation, MS, elementary analysis), analysing the data, storing, writing up, discussion of data obtained.”

When respondents described an experiment they consistently used the elements of the traditional understanding of scientific method. Common terms included: hypothesis, comparison, measurement, treatment, control, and analysis. However, a notable omission across all respondents was that none of them included any form of conclusion step in their definitions.

A bigger variation in the responses came in the unit of analysis. Six responses mentioned that an experiment is a series of trials, whereas five responses described a single trial as an experiment. The remainder of responses either did not mention a unit of analysis or were left blank. These definitions were used to analyze the publication and replication questions described in the next two sections. Unfortunately, the divergence over unit of analysis complicated our analysis for the next set of questions, because without a shared definition of the scope of what constitutes a single experiment, responses on the percentage of experiments that are or should be published/replicated cannot be directly compared.

#### Publications

Q-6 asked what proportion of the experiments conducted in the respondent's field end up in a publication. Table 3 shows the distribution of responses.

**Table 3:** Percentage of Experiments Conducted that are Published.

Percentages	Number of Respondents
0–19	6
20–39	3
40–59	2
60–79	1
80–100	2

Distribution of responses to question Q-6 “To your best approximation, what percentage of the experiments you conduct end up in published work?”.

Aggregating these responses suggests that overall, fewer than half of all experiments are published. However, the most notable aspect of this result is that respondents are spread across the entire range of possible answers, indicating a wide diversity of experience over what gets published.

We also analyzed these responses with respect to the unit of analysis given by each respondent in their definitions of an experiment (Q-5). Those respondents who defined an experiment as a *series of trials* gave answers to Q-6 in the range of 5–59%. Respondents who defined an experiment as a *single trial* gave answers in the 5–39% range. Respondents who did not specify a unit of analysis in their definition of an experiment gave answers between 60–100%. However, our sample size is not sufficient to draw any conclusions from this distribution, other than to note that the three highest estimates came from respondents who were the least clear about what constitutes an experiment.

#### Replications

Questions 7 and 8 asked about replication – what proportion of experiments (in your field) *are* replicated and what proportion *should be* replicated. Overall, no respondents indicated that more than 60% of experiments conducted in their field were replicated, and the majority said less than 20% are replicated. Some of the respondents gave two answers: one percentage for replications by researchers in the same lab and a second response for external replications. In these cases, we have included only the answer for external replication in table 4.

**Table 4:** Percentage of Experiments Replicated and Should be Replicated.

	Number of Respondents	
Percentages	% of expts ARE replicated	% of expts SHOULD BE replicated
0–19	9	4
20–39	3	1
40–59	2	1
60–79	0	0
80–100	0	2

Distribution of responses to question Q-7 “To your best approximation, what percentage of the experiments conducted in your field are replicated?” and Q-8 “In your opinion, what percentage of the experiments conducted in your field should be replicated?”.

Participants used the open format of the response form to add further insights. Some described problems with replications:

“probably replication is attempted 10% of the time, probably successfully 0% of the time.”

Two respondents differentiated between replications done within the same laboratory versus replications done outside the laboratory where the original result was found:

“Don't know. Depends on meaning of “replication”. If it means repeated by same researchers, 90%. If it means by others, perhaps 5%?”

The original intent of the question was to ask about independent replications, and hence, in hindsight, the wording was ambiguous. Detailed analysis of the freeform responses shows that only one respondent may have interpreted the question other than as intended.

Another respondent raised the question of *scooping* and *secrecy* here (which are explicitly addressed in later questions):

“Depends what you mean by replicated. If you mean directly repeated by different hands, maybe around 30–40% – mainly because everyone keeps their results secret so things are needlessly repeated. If you mean experimentally confirmed, in the long run that approaches 100% because unreliable results are found out by subsequent [experts].”

This indicates another distinction - experiments that are deliberately replicated versus those that are ‘inadvertently’ replicated.

On the question of how many experiments *should be* replicated, respondents disagreed widely. Although there is only a small change between the counts of the two columns of Table 4, there is reduction in total count for the second column, as only eight of the nineteen respondents volunteered percentages for this question. Four left the question blank, and two indicated that they had no idea. One interpretation of the low response rate for this question is a lack in the confidence in the participants' responses.

The detailed responses revealed some interesting differences of opinion. One respondent declared that anything published should be replicated, while another implied that all replications should have purpose. In a similar vain, three responses recommended that all *important* experiments should be replicated. For example,

“[i]nteresting or important experiments should all be replicated. Boring ones can go quietly to their death.”

Most respondents provided a comment explaining their belief. One respondent believed everything important should be replicated and that communicating results is important:

“…scientists being able to offer additional comments, even if there were comments possible on papers much like blog comments.”

Furthermore, two respondents opposed replications:

“I mean, science relies upon the fact that each investigator is telling the truth. Rather than wasting efforts double checking each other constantly, there probably only need to be spot checks–enough to keep people honest…. Even with the current low replication rate, I feel like the vast majority of published experiments are legitimate and accurately portrayed in the literature.”

“If we didn't waste so much time playing Spy vs Spy, and exchanged information in real time, probably only about 10%, really crucial results, would need to be directly repeated.”

Finally, another theme that arose was the idea of natural replication:

“Replication seems to happen naturally; when someone wants to extend your work, they start by replicating your published work as a first step (validation).”

The wide range of responses to this question illustrates that the role and significance of replications is still up for debate with in the scientific community.

### Peer Review

Q-9 through Q-12 explored the peer review process for peer reviewed journals. Q-9 asked the participants to evaluate the error rates for various criteria in a typical peer reviewed journal in their field. Q-10 asked them to describe how the peer-review process works in their field, while Q-11 and Q-12 asked about benefits and drawbacks of peer-review. Responses show that the respondents' views on how peer-review works were similar, and all of them agree that peer-reviewed journal papers often contain errors. The benefits (Q-11) focussed on improvements to the quality of the paper and the maintenance of the standard of the journal. The drawbacks (Q-12) were associated with the mechanics of peer-review; for example many respondents took issue with anonymity of the reviewers.

Table 5 summarizes the responses to Q-9, giving the responses for each category of error. All respondents believe that peer review journals contain errors: no respondent gave a figure less than one percent in any of the error categories, but the majority of respondents indicated that errors in each category affect less than 10% of journal papers (Q-9).

**Table 5:** Error Rates in Peer Reviewed Journals (Number of Responses).

Percentage	(A) Incorrect Citations	(B) Incorrect Figures	(C) Incorrect Facts	(D) Flawed Results
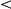 1	0	0	0	0
1–4	3	3	3	2
5–9	5	8	5	5
10–19	2	1	1	1
20–39	2	0	4	1
40–59	1	0	0	3
60–79	1	2	0	0
80–100	0	0	1	2

Distribution of responses to question Q-9 “To your best approximation, what percentage of papers published in a typical peer reviewed journal in your field (in the past year) contains: (A) incorrect citation (B) incorrect figures (C) incorrect facts (D) flawed results.”

Respondents were generally confident in the overall quality of peer reviewed scientific journals, even though they know these journals contain errors. For the fourth category, flawed results, the median answer is that *one in ten* papers published in a peer review journal contain flawed results, although several respondents thought *around half* of all papers do, and a couple thought that *more than 80%* of all papers do.

The *descriptions* of the peer review process given in response to Q-10 were consistent. Their descriptions included:

- a section editor who would receive submissions
- reviewers who would be selected and sent submission for review
- reviewers who would submit comments/feedback/revisions
- a section editor who would make the final call

Variations included the number of reviewers (responses varied between two and four) and whether journals allow the author to suggest appropriate reviewers. Some respondents indicated that some journals are associated with a conference where the author would present.

However, the participants' *opinions* of this process differed markedly. To assess the respondents' opinions of the peer-review process, we coded the responses according to emotional tone. One response was coded positive, seven responses were coded neutral, four responses were coded slightly negative, three responses were coded very negative. Four responses were blank.

A sample neutral response:

“A researcher submits work to a journal and then it is passed on to two or three reviewers who make comments and accept, accept with changes, or reject the work”

A sample negative response:

“[Manuscript] sent to journal, editor farms it out to 2 or 3 peers, wait several months, get back one review, editor decides accept/reject based on what they had for breakfast that morning.”

Respondents are consistent in their opinions of the benefits and drawbacks of peer review (Q-11, Q-12). We used an iterative coding strategy to analyze participants answers to these two questions (similar to the process described above for Q-3 and Q-4). We then used an iterative clustering process to compare the benefit/drawbacks. Two clusters emerged: *improving the quality of the paper* and *maintaining the standard of the journal*. The benefits identified with each cluster are shown in Table 6.

**Table 6:** Benefits of Peer Review.

Paper Quality	Journal Standard
find errors, improve language elements, constructive criticism, second eyes, identify related work, fact checking, comments, and suggestions	weed out unimportant work, minimize bad research, maintain scientific merit, reduce frequency of publication, and quality filter

Major emergent axes (*paper quality* and *journal standard*) as benefits of the peer review process as discussed by the respondents (Q-11).

For drawbacks, we identified the following representative terms: reviewer anonymity, turn around time for submissions, lack of discussion, demand for new experiments, competition, lack of reviewer availability, pushing trendy work, and suppressing innovation. Only one participant responded that there are no drawbacks to the peer review process.

Some of the drawbacks that were mentioned are tied to the benefits, specifically maintaining the standard of the journal. For example, competition and a demand for new experiments are drawbacks for the individual researcher, but may help to raise the quality of the journal. Other drawbacks appear to be the result of the process itself. Several participants talked about the time it takes to publish and the lack of communication between editors and authors. One respondent offered the following advice to mitigate the problem with the length of time between submission and publication:

“With several journals allowing pre-prints of various types the wait is a non issue. We have published work on blogs, wikis, pre-print archives, etc. while waiting for the final manuscript to be peer-reviewed.”

The remainder of the process-oriented drawbacks are centred around the reviewer and the reviewer's anonymity. Participants argued that since the reviewers are anonymous it allows them to be biased and to push their personal agenda or work. They indicated that the reviews seem arbitrary and that since there is no discussion or no medium to respond to the review there is no way to defend their claim. Participants discussed how it is hard to find reviewers who have time and are knowledgeable, without a conflict of interest; and how this results in poor reviews. As one respondent put it:

“[g]ood reviewing is the province of the rich in time and those with no inferiority complex.”

### Publications, Data and Results Sharing

We found consistent views on data and results sharing within our sample. The respondents indicated that publications, data, and results should be shared with everyone and should be available to the public for free. However, they disagreed on the time at which the data and results should become available. These responses ranged between “as soon as possible” and “after publication”. Many different reasons were given on why scientist don't share, with no overall consensus on these.

#### Sharing

Questions Q-13 through Q-16 dealt with the scientists' views concerning to whom and under what circumstances their publications, supporting data, and results should be shared, while Q-17 asked when the supporting data and results should be shared. In the following discussion, each question had fourteen respondents.

All those who responded indicated that that everyone should have access to their publications (Q-13). And all indicated that access to their publications should be free to the reader (Q-14). One respondent went so far as to say that the publication cost should fall upon the author.

When asked who should have access to the scientist's data and results, all those who responded indicated that everyone should have access (Q-15). Two respondents add the caveat that it should be available only upon request. Another two respondents add that this information should only be available after publication. All responses showed that data and results should be available at no cost (Q-16). One response added that cost associated with data storage should fall on the home institution.

The major disagreement was to do with *when* data and results should be shared. Table 7 shows the response to Q-17 “What point in time should your data and results be available?”. *As soon as possible* is difficult to interpret as an answer. The following is one interpretation:

“As soon as possible. Preprint servers like Nature Proceedings and arXiv can make pre-review information available, and peer review can be used as a first-pass filter on top of that.”

**Table 7:** Time frame for Data and Results Availability.

Time	Count
As Soon As Possible	4
After Review	1
After Publication	8
Within Reason	1

Distribution of responses to questions Q-17 “What point in time should your data and results be available?”.

*After Publication* was the most common response. This indicates that once results are made public through a publication, the desire to protect the supporting evidence is less important to the original research team. Respondents discussed web-links between their publications and the data.

The response *Within Reason* included a suggestion that access to the work should be independent to the state of the publication process:

“Incrementally, but not only after publication. Daily results don't need to be posted, but science should be made available within, say, a month or two after the work is completed, whether a paper is published or not.”

Analysis of these responses leads us to suggest that scientists can be categorized into four groups:

1. Those who share their data and results immediately.
2. Those who share their data and results eventually.
3. Those who believe in sharing data and results, but who do not share them due to limiting factors (such as concerns over scooping and/or publisher restrictions).
4. Those who do not believe in sharing data and results (beyond what is included in their published papers).

We did not have any respondents from the final category, but this was to be expected, given that our sample is drawn from scientists most closely identified with initiatives to promote various approaches to open science. Further research is needed to assess what proportion of scientists would fall into the each category.

#### Limiting Factors

The last three questions focussed on factors that limit scientists from sharing publications, data and results. For the responses to Q-18, on factors that limit sharing, we identified the following representative terms: time pressure, patent pressure, publication pressure, publisher's restrictions, infrastructure issues, and scooping. Patent pressure and scooping appear to be the major concerns, as they were each mentioned five times.

Q-19 asked specifically about scooping. The responses indicated a range of experiences with scooping, which we grouped into three different types: non-malicious scooping, malicious scooping, and copyright infringement. Six respondents mentioned being scooped in the past. Four of these were the result of non-malicious scooping where two teams were working on the same research independently. For example:

“I've had people publish results similar to mine, but that didn't affect my own ability to publish.”

“Worked [for] half a year on a project that was published about 6 months down the line.”

One respondent shared an experience of malicious scooping, where work was deliberately taken and used by someone else:

“Yes. Presented computational work as a poster at a meeting which was nominally closed. Saw the same work presented by people who were at that meeting 1–2 years later…. Should have published sooner but didn't due to teaching pressures. At least I knew it was quality work, but since it was my first paper (on my own), it took a while for me to get confidence to publish anything afterwards.”

The final type of scooping discussed was use without citation (copyright infringement):

“[Y]es, [I] put a lot of material on the web and know that sometimes other people (non-scientists) use it in their materials (though not for scientific publication) without credit[.]”

Respondents were mainly concerned with malicious scooping as it is more likely to impact their career, yet only one respondent was actually affected by it.

The final question, Q-20, asked about publishers' restrictions. Three respondents indicated that they have had problems publishing because some of their data or results had appeared previously on the internet. In the first case the authors had to remove the material that had appeared, in the second case the author found another publisher who would accept it, and in the third case the author created a new venue to publish his work. Two respondents also noted being very fearful of this issue.

## Discussion

This study set out to explore the views and motivations of those involved in a number of recent and current advocacy efforts aimed at making science and scientific artifacts accessible to a wider audience. These advocacy efforts build on a long-standing concern over science literacy and the increasing gulf between sciences and the public discourse. Recognition that such a gulf exists dates back at least as far as C. P. Snow's 1959 Rede Lecture on “The Two Cultures”, in which he argued that scientists and “literary intellectuals” increasingly are unable to comprehend one another [10]. He further claimed that scientists were required to have basic knowledge in the humanities, but non-scientists were not required to have a basic knowledge in science. The Rede Lecture caused a significant debate within the intellectual community, with strong arguments both for and against his views.

The divide seemed to explode into full blown “science wars” in the 1990's, with the Sokal affair, in which a physicist had a hoax paper accepted in a cultural studies journal *Social Text*, leading to a war of words between scientists and those who questioned scientific objectivity. However, as Sokal himself has pointed out [11], the affair is much more about sloppy standards of work and sloppy journal editors than it is about a cultural war. Concerns about the integrity of the peer review process were stoked even more by a series of high profile retractions in the last decade, including the Bogdanov affair [12], in which a series of papers published in reputable theoretical physics were discovered to be nonsense (although there is still an open question on whether they were a deliberate hoax or just shoddy work); and the Schön scandal, in which a whole series of papers on molecular semiconductors in prestigious science journals had to be retracted as fraudulent [13].

Part of the problem in interpreting the significance of these events seems to be a misunderstanding about the role of peer review. Many authors describe the peer review process as ‘a sacred pillar’ of science, and point to its failings as a symptom of deeper problems over the practice of science (see for example Horrobin [14], who argues that peer review stifles innovation). However, our respondents presented a much more nuanced view of peer-review, as a messy process, which often allows papers to be published that contain errors and flawed results, but which also brings many benefits in improving papers prior to publication and in maintaining the overall quality of each journal. Scientists understand that it's a flawed process, but those outside of science continue to be shocked when it's shown to be flawed.

The bigger issue then, is the perception of science by the public, and especially the way that it is portrayed in the media. In his recent book, Unscientific America, Chris Mooney charts the decline of public understanding of science, particularly in the US [15], and the decline of coverage of science in the media. For example, the 2008 report on the state of news media reported that for every five hours of cable news only one minute of news coverage was on science and technology [16]. In print media, there has been a dramatic reduction in the number of science sections. In 1989, 95 newspapers featured weekly science sections. This number fell to 44 by 1992 [17]. Miller found in 2004 that only 17% of American adults were able to comprehend *The New York Times* science section, which he used as a qualification of basic scientific literacy [18]. The scientists in our study recognize that there are problems in the way science is communicated (many of them cited specific instances of misrepresentation of science in the media), and all agreed that public understanding of their own fields, and of science in general, is lacking.

With this context, the advocacy efforts we explored in this study can be seen as different responses to address these problems. These responses span a number of distinct goals, including attempting to reduce the incidence of erroneous results (e.g. by sharing more of the intermediate steps of scientific research), improving the ability of scientists to replicate one another's work (e.g. by setting standards for reporting details of procedures used), and improving public understanding of and engagement in science (e.g. by opening up the process of doing science to outsiders). In surveying the opinions of scientists involved in these initiatives, we expected to find a great deal of common ground, in terms of the challenges they face, and the overall goals of their advocacy efforts. Although some areas of consensus emerged, the divergence of opinion on some questions was stark, leading us to revisit three of these initiatives (computational provenance, reproducibly research, and open science) to seek to explain the divergence.

### Computational Provenance Advocacy

“Computational Provenance” was the theme of the May/June 2008 issue of IEEE's *Computing in Science* & *Engineering*. Computational provenance focuses on data and its history, asking questions about who created/modified the data and the results it produced, and when that happened. Computational Provenance is a systematic way of capturing this information [19].

The provenance movement has advanced though three provenance challenges, organized around competitions to develop a work flow tracking tool. The first challenge in the summer of 2006 brought diverse results and discussion, which prompted the later challenges. The second provenance challenge led to the proposed specification of a data model and inference rules entitled the Open Provenance Model (OPM). The OPM provides a core representation of information to be collected about provenance for such workflow systems. The third provenance challenge resulted in changes to the OPM specification and additional profiles for OPM [7].

Freire et. al. provide a survey of provenance-enabled systems, and describe the features of provenance management solutions. The three main components of a provenance-enabled system are a capture mechanism, a representational model, and an infrastructure for storage, access, and queries [20]. When these three components are effectively combined they allow for tracking both data and data history.

Miles et. al. discuss how provenance can affect the scientific work flow and vice versa. He discusses the use of the workflow tool Pegasus and concludes:

“Understanding the process that ultimately produced a result is critical to correctly interpreting it, and it's particularly important when execution steps aren't apparent in the original process design. Provenance is a key ingredient of scientific reproducibility: colleagues can share this type of information and thereby reproduce and validate each other's results.” [21]

The efforts of computational provenance advocates are very focused. The work concentrates specifically on digital information (e.g. scientific datasets) and the processing steps that transform such data. Descriptions of this work mention reproducibility as a goal but do not focus on it; instead the overall aim is to make the processing steps more transparent, and to provide a traceable history for each dataset. The lack of an explicit focus on reproducibility within this initiative might go some way to explaining the diversity of opinions on what proportion of experiments should be reproduced.

### Reproducible Research Community

Reproducible Research is another suggestion to improve the credibility of current scientific research. This community has not yet been thoroughly discussed in the literature, however, it has been receiving more attention of late. Most notably, “Reproducible Research” was the theme of the January/February 2009 issue of IEEE's *Computing in Science* & *Engineering*. In the introductory article, Fomel argues that:

“The success and credibility of science is anchored in the willingness of scientists to expose their ideas and results to independent testing and replication by other scientists. This requires the complete and open exchange of data, procedures, and materials.” [5]

This statement summarizes the perspective of many reproducible research advocates. Also in the same issue, Donoho et al. discusses their view on the current credibility crisis, indicating that empiricism, deduction, and computation is highly error-prone.

“Vagueness, wandering attention, forgetfulness, and confusion can plague human reasoning. Data tend[s] to be noisy, random samples contain misleading apparent patterns, and it's easy to mislabel data or misinterpret calculations.” [22]

The development within the research community has so far been a grassroots approach, with the majority of scientists publishing their views and research on their personal websites and web-forums [23]. For example, Donoho has created a family of Matlab extensions to support reproducibility [22], while Stodden has created a proposed legal framework, the Reproducible Research Standard, which sets out the requirements for sharing the detailed information that supports reproducibility [24].

These two efforts are examples of the reproducible research advocacy community's growth and its diversity. While much of the effort needed to achieve the kind of reproducibility discussed would include accurate capture of provenance, there appears to be little overlap between the two communities. The reproducible research community has adopted a broader scope, but has been less focused in their efforts, despite having clearer goals.

### Open Science Community

Open science advocates urge scientists to make scientific artifacts more readily available to broad audiences, using standard web technologies. The core philosophy is to approach scientific research in the same manner as open source software projects: all steps of the research should be visible to anyone who cares to see them. The emphasis is placed squarely on public availability and accountability, although support for better collaboration between scientists (e.g. by improving reporting practices to support reproducibility) are included as further justification for more openness. One exemplar is the Open Science Project [25], which has the goals:

“Transparency in experimental methodology, observation, and collection of data.

Public availability and reusability of scientific data.

Public accessibility and transparency of scientific communication.

Using web-based tools to facilitate scientific collaboration.” [25]

These goals would allow for communicating a full account of the research at all stages. Advocates of open science believe that the academic incentive structure (money, reputation, and promotion) encourages “closed” research. These advocates are attempting to address this and to change the way science productivity is measured [25].

The open notebook science community, a subset of the open science community, takes this one step further by advocating the publication of their laboratory notebooks online. Bradley and Neylon discuss this approach in *Nature News* [26]. They explain that putting their lab notebooks online forces a higher standard upon them, because outsiders can view every step. They also claim it enables them to get feedback and find errors in their work much faster. The open notebook science community also runs a challenge, with awards to encourage scientists to make their notebooks and results available. They have created branding logos to indicate scientists who are following the challenge [6].

### Implications

The most striking result from our study is the lack of consensus among the study participants on several concepts that are central to these initiatives. For several of the questions, the responses varied across the full range of possible answers:

- Percentage of experiments that become published;
- Percentage of experiments that should be replicated;
- Percentage of papers that contained flawed results (although nobody said zero);
- Opinion on how well the peer review process works (ranging from positive to very negative opinion);
- Point at which data and results should be released (ranging from ASAP to after publication, although nobody said ‘never’)

In designing and conducting the study, a number of colleagues and even some of the study participants remarked that our results would not be credible because the sample was not representative of the entire scientific community–being collected from conferences emphasizing new technology, with speakers from the various advocacy groups discussed above. However, our study was specifically designed to explore opinions within this sub-community, as opposed to generalizing our findings within the broader population of experimental scientists. Instead, we have focussed on the diversity of views within our sample, and the lessons that can be drawn about a lack of consensus over key concepts. But this focussed sampling makes the results even more surprising; we expected to to see a consensus on many of these questions, indicating a shared set of goals in making scientific research more accessible.

For these various advocacy groups to be effective they need to develop a stronger internal consensus on these questions, as a prelude to developing a broader consensus across the scientific community on how to improve things. Further study is required to understand the reasons for this diversity of opinion within the advocacy community.

The results on error rates for incorrect citations, incorrect figures, incorrect facts, and flawed results are also worth noting. All respondents recognized that such errors occur in a non-negligible subset of published papers, even though they differed on the extent of the problem. Errors in published works are sometimes corrected in followup correspondence, but our experience is that this happens a lot less often than the error rates reported by our participants would suggest is needed. The key insight here is that such errors and flawed results become tacit knowledge in the specific research community. Small errors that do not affect the outcome of a work usually go uncorrected, while some are addressed through errata. More often, the correction only appear in correspondence or later papers that discuss the work, which means that only the scientists in the field with extensive knowledge of the literature are likely to know about them. None of the initiatives discussed above directly address this problem, and it is indeed a barrier for broader accessibility to scientific results. We suggest that further research is needed into mechanisms to link corrections more directly with the original papers, and further study is needed of the ratio of paper publication to comment/errata to explore how often published work is subsequently corrected in different fields of science.

Although the participants differed on their opinions of how well the peer review process works, the majority of them gave relatively negative assessments, and together they listed a large number disadvantages of the process. One drawback mentioned was the assumption of correctness in peer review journals. In Kronick's historical look at the peer review process, he discusses the link between intellectual societies and peer review [27]. Arguably peer review was invented by exclusive societies to maintain their credibility. If very prestigious societies and journals adopted peer review first, and other less prestigious journals then followed suit; then it is likely that peer review is more of a symbol of the (aspirational) status of a journal, rather than a mechanism to ensure correctness of the papers it publishes.

The emphasis on peer reviewed publications also neglects another problem, noted by Goetz [28]: that unpublished research remains hidden from view, along with the data and results that it produces. Over-emphasis on peer review publication as the main mechanism for disseminating the results of scientific work means that many of the useful insights produced by failed studies goes to waste, along with all the data they produce.

This analysis suggests that the various initiatives described above have all correctly diagnosed the problem - that peer review needs to be supplemented with a number of other mechanisms that help to establish the correctness and credibility of scientific research and to disseminate the intermediate products of that research. However, they differ widely in their opinions over the nature of what mechanisms are needed. We suggest a broader discussion is needed of the goals of these various initiatives, to develop a larger consensus across the scientific community.

### Limitations

In designing this kind of study, there is a natural trade off between in-depth analysis of a small number of respondents, and a broader, but shallow survey of a larger, representative sample. We chose the former approach for this work because there is no prior literature that investigates the key concepts, and we wanted to investigate in depth how members of the open science movement characterize their aims.

The limitations of this study lie primarily in the selection bias of the respondents and the repeatability of the analysis process.

We anticipated a slightly higher response rate than what was achieved. More participants may have allowed us to assess in more detail how much heterogeneity there is across this community. Hence our results should be taken as preliminary evidence of a lack of consensus–further research is needed to establish whether this result is robust.

We collected basic demographic information (Q-21 to Q-27) and information about the respondent's field (Q-1, Q-2). We did not manage to recruit subjects from all of the major fields outlined in the questionnaire. Furthermore our subjects turned out to be drawn from a very limited demographic. Most respondents were between 30–49 years of age and were male. All respondents were from North America or Europe. Almost all worked in academia and a significant fraction (about 40%) were PhD students. We had anticipated that more professors than students would submit responses.

The intended target population for the questionnaire was experimental scientists. Because of the necessary anonymity for human subject ethics approval required in administering the questionnaire, it is impossible to determine whether all the respondents were indeed from our target population of experimental scientists associated with the various initiatives around making science more accessible. However, the passion and detail of the responses shows that all respondents found the topic personally relevant, increasing our confidence that our sampling strategy was relatively successful.

Since we collected the data through a questionnaire, we did not have prolonged contact with the respondents, so we were unable to confirm our interpretation of the responses with the respondents.

As with all qualitative analysis, our coding strategies include a fair amount of subjective judgment. To mitigate this, throughout the analysis process we used peer debriefing to verify that the codes generated were appropriate. An internal colleague, with training in qualitative methods, reviewed our methodology and the results we showed, and was given access to all our data and notes.

### Conclusions and Future Work

This study explored the opinions of scientists involved in various advocacy efforts that are aimed at increasing access to the products of scientific research. We probed the views of these scientists on the topics of reproducibility, credibility, scooping, data sharing, results sharing, and the effectiveness of the peer review process.

Our key findings were:

- respondents perceive that the general public is ignorant of most aspects of science;
- respondents perceive that public opinion of science varies depending on the field of science under discussion, with fields personally relevant to the public rated higher;
- although common terminology was used when defining what constitutes an experiment, there were large variations over the unit of analysis. Such variations make it hard to compare opinions on questions to do with what proportion of experiments are or ought to be replicated;
- opinions varied widely on what percentage of experiments should be replicated (even after allowing for divergent definitions of ‘experiment’). This reflects a variety of opinion on what constitutes replication, and indeed, a variety of opinion on the role and value of replication in science;
- respondents reported consistent descriptions of the peer review process, but differed widely on their assessment of how well it works;
- respondents described a variety of benefits and drawbacks of peer review. The benefits can be categorized as things that either improve the quality of the paper or maintain the standard of the journal. The drawbacks can be categorized as resulting from the process, resulting from reviewer anonymity, or occurring as a side effect of the benefits.
- all respondents believe that publications, raw data, and results should be available to everyone free of charge, but differed in their opinions on when this should happen within the scientific process and who should bear the cost;
- scooping and patent pressures were the most commonly cited factors preventing scientists from making their publications, raw data, and results more widely available.

This study was a preliminary investigation of these issues, and further work is needed to explore and characterize the nature of the lack of consensus apparent in our results. In particular, further studies are needed to establish how the results of this study compare to the opinions of a broader cross-section of experimental scientists. In follow-up studies, we hope to explore three additional research paths:

- Understand the perceptions of the public on the credibility of scientific research and their interest in it. We will ask questions such as: How do you define good science? How do you define credible science? Which fields of science are favoured, and why?
- Understand the interaction of the goals of the stakeholders (funders, publishers, academic institutions, governmental policy makers, scientists, and the general public) with respect to accessibility of scientific work, and how these stakeholders will impact and be impacted by changes in science communication.
- Understand the differences between the groups of scientists (early adopters, trend followers, and skeptics) with respect to scientific communication.

## Supporting Information

Appendix S1 **Questionnaire Questions.** List of the questions that were included on the respondents questionnaire.
(PDF)

## References

[1] Fjällbrant N (1997). Scholarly communication – historical development and new possibilities..

[2] McGuigan GS, Russell RD (2008). The business of academic publishing: A strategic analysis of the academic journal publishing industry and its impact on the future of scholarly publishing.. Electronic Journal of Academic and Special Librarianship.

[3] Hendler J (2007). Reinventing academic publishing-part 1.. IEEE Intelligent Systems.

[4] Willinsky J (2003). The nine avours of open access scholarly publishing.. Journal of Postgraduate Medicine.

[5] Fomel S, Claerbout JF (2009). Guest editors' introduction: Reproducible research.. Computing in Science & Engineering.

[6] Unknown (2009). Open notebook science challenge.. http://onschallenge.wikispaces.com/.

[7] Unknown (2009). Provenance challenge.. http://twiki.ipaw.info/bin/view/Challenge/.

[8] Strauss A, Corbin J (1998). Basics of Qualitative Research.

[9] Rutherford A (2008). Palin and the fruit fly.. http://www.guardian.co.uk/commentisfree/2008/oct/27/sarahpalin-genetics-fruit-flies.

[10] Cornelius DK, StVincent E (1964). Cultures In Conict: Perspectives on the Snow-Leavis Controversy..

[11] Sokal AD (1996). A physicist experiments with cultural studies.. Lingua Franca.

[12] Baez J (2006). The Bogdanoff Affair.. http://math.ucr.edu/home/baez/bogdanoff/.

[13] Reich ES (2009). The rise and fall of a physics fraudster.. Physics World May.

[14] Horrobin DF (1990). The philosophical basis of peer review and the suppression of innovation.. The Journal of the American Medical Association.

[15] Mooney C, Kirshenbaum S (2009). Unscientific America: How Scientific Illiteracy Threatens Our Future..

[16] Unknown (2008). The state of the news media 2008: An annual report on american journalism.. http://www.stateofthemedia.org/2008/narrative_cabletv_contentanalysis.php?cat=1&media=7.

[17] Russell C (2006). Covering controversial science: Improving reporting on science and public policy..

[18] Miller JD (2004). Public understanding of, and attitudes toward, scientific research: What we know and what we need to know.. Public Understanding of Science.

[19] Silva CT, Tohline JE (2008). Computational provenance.. Computing in Science & Engineering.

[20] Freire J, Koop D, Santos E, Silva C (2008). Provenance for computational tasks: A survey.. Computing in Science & Engineering.

[21] Miles S, Groth P, Deelman E, Vahi K, Mehta G (2008). Provenance: The bridge between experiments and data.. Computing in Science & Engineering.

[22] Donoho D, Maleki A, Rahman I, Shahram M, Stodden V (2009). Reproducible research in computational harmonic analysis.. Computing in Science & Engineering.

[23] Unknown (2009). Reproducible research.. http://reproducibleresearch.net/index.php/Main_Page.

[24] Stodden V (2009). The legal framework for reproducible scientific research: Licensing and copyright.. Computing in Science & Engineering.

[25] Gezelter D (2009). The open science project.. http://www.openscience.org/blog/.

[26] Bradley JC (2008). Data on display..

[27] Kronick DA (1990). Peer review in 18th-century scientific journalism.. The Journal of the American Medical Association.

[28] Goetz T (2007). Freeing the dark data of failed scientific experiments.. Wired Magazine.

