# Supplementary material for: On the Lack of Consensus over the Meaning of Openness: An Empirical Study
Source: PLoS One. 2011 Aug 17;6(8):e23420. doi: 10.1371/journal.pone.0023420 (PMC3157385; doi:10.1371/journal.pone.0023420)
Supplement: Appendix S1 — Questionnaire Questions. List of the questions that were included on the respondents questionnaire. (PDF) [file pone.0023420.s001.pdf]

# Appendix S1: Questionnaire Questions

The following are the questions that were included on the questionnaire.

Q-1 Select the research field, from the list below that is the closest resemblance of your own:
Applied Science / Astronomy / Biology / Chemistry / Earth Science / Environmental Science / Life Science / Medicine / Physics / Psychology / Other

Q-2 Provide more details about what your particular field of research is?

Q-3 How do you think the general public (non-scientists) views your particular field's research efforts?

Q-4 How do you think the general public (non-scientists) views the efforts of the scientific community as a whole?

Q-5 Characterize what constitutes an experiment in your field. If you don't use this term what term and meaning would be the closest.

Q-6 To your best approximation, what percentage of the experiments you conduct end up in published work?

Q-7 To your best approximation, what percentage of the experiments conducted in your field are replicated?

Q-8 In your opinion, what percentage of the experiments conducted in your field should be replicated?

Q-9 To your best approximation, what percentage of papers published in a typical peer reviewed journal in your field (in the past year) contains:
(A) incorrect citation (B) incorrect figures (C) incorrect facts (D) flawed results

Q-10 Discuss the peer review process, for a typical journal in your field.

Q-11 What are the benefits of the peer review process?

Q-12 What are the drawbacks of the peer review process?

Q-13 Who should have access to your publications (e.g. reviewers, teachers, government, scientist, general public)?

Q-14 Under what circumstances and cost should your publications become available (e.g. free, at cost, yearly fee, availability at libraries, by request)?

Q-15 Who should have access to your research data and results (e.g. reviewers, teachers, government, scientist, general public)?

Q-16 Under what circumstances and cost should your research data and results become available (e.g. free, at cost, yearly fee, availability at libraries, by request)?

Q-17 What point in time should your data and results be available (e.g. as soon as possible, after publication, after review)?

Q-18 What limiting factors do you have with regards to availability of your publications, data, and results (e.g. scooping, patents, publications pressures)?

Q-19 Have you ever been "scooped"? If so, tell us about it.

Q-20 Have you ever had problems publishing you work because your data/results appeared previously on the web? If so, tell us about it.

Q-21 Age?
<20 / 20-29 / 30-39 / 40-49 / 50-59 / 60-69 / 70-79 / 80+

Q-22 Gender?
Male / Female / Other

Q-23 Country of Residence?

Q-24 In what country did you complete your highest degree?

Q-25 Position?
Lab Technician / Junior Researcher / Senior Researcher / PhD Candidate / Postdoctoral Researcher / Assistant Professor / Associate Professor / Professor / Other (please specify)

Q-26 What type of laboratory do you work in?
Academic / Government / Private / Other (please specify)

Q-27 To your best approximation, how many publications are you an author on in a peer reviewed journal or conference?
